# OCIAD1 is a host mitochondrial substrate of the hepatitis C virus NS3-4A protease

**DOI:** 10.1371/journal.pone.0236447

**Published:** 2020-07-22

**Authors:** Huong T. L. Tran, Kenichi Morikawa, Anggakusuma, Rose Zibi, Viet Loan Dao Thi, François Penin, Markus H. Heim, Manfredo Quadroni, Thomas Pietschmann, Jérôme Gouttenoire, Darius Moradpour

**Affiliations:** 1 Division of Gastroenterology and Hepatology, Lausanne University Hospital and University of Lausanne, Lausanne, Switzerland; 2 Institute for Experimental Virology, TWINCORE Centre for Experimental and Clinical Infection Research, Hannover, Germany; 3 Institut de Biologie et Chimie des Protéines, Bases Moléculaires et Structurales des Systèmes Infectieux, UMR 5086, CNRS, Labex Ecofect, University of Lyon, Lyon, France; 4 Division of Gastroenterology and Hepatology, University Hospital Basel, Basel, Switzerland; 5 Protein Analysis Facility, University of Lausanne, Lausanne, Switzerland; Centre de Recherche en Cancerologie de Lyon, FRANCE

## Abstract

The hepatitis C virus (HCV) nonstructural protein 3-4A (NS3-4A) protease is a key component of the viral replication complex and the target of protease inhibitors used in current clinical practice. By cleaving and thereby inactivating selected host factors it also plays a role in the persistence and pathogenesis of hepatitis C. Here, we describe ovarian cancer immunoreactive antigen domain containing protein 1 (OCIAD1) as a novel cellular substrate of the HCV NS3-4A protease. OCIAD1 was identified by quantitative proteomics involving stable isotopic labeling using amino acids in cell culture coupled with mass spectrometry. It is a poorly characterized membrane protein believed to be involved in cancer development. OCIAD1 is cleaved by the NS3-4A protease at Cys 38, close to a predicted transmembrane segment. Cleavage was observed in heterologous expression systems, the replicon and cell culture-derived HCV systems, as well as in liver biopsies from patients with chronic hepatitis C. NS3-4A proteases from diverse hepacivirus species efficiently cleaved OCIAD1. The subcellular localization of OCIAD1 on mitochondria was not altered by NS3-4A-mediated cleavage. Interestingly, OCIAD2, a homolog of OCIAD1 with a cysteine residue in a similar position and identical subcellular localization, was not cleaved by NS3-4A. Domain swapping experiments revealed that the sequence surrounding the cleavage site as well as the predicted transmembrane segment contribute to substrate selectivity. Overexpression as well as knock down and rescue experiments did not affect the HCV life cycle *in vitro*, raising the possibility that OCIAD1 may be involved in the pathogenesis of hepatitis C *in vivo*.

## Introduction

Hepatitis C virus (HCV) infects about 70 million people and represents a major health burden worldwide [1]. Direct acting antivirals now allow to cure the majority of those affected but challenges in basic, translational and clinical research remain [2, 3]. HCV contains a 9.6-kb positive-strand RNA genome encoding a polyprotein that is co- and posttranslationally processed by cellular and viral proteases [4]. The nonstructural protein 3-4A (NS3-4A) protease is a key component of the viral replication complex and the target of protease inhibitors used in current clinical practice. By cleaving and thereby inactivating selected host factors it also plays a role in the persistence and pathogenesis of hepatitis C [5, 6]. Indeed, it has been shown to suppress host innate immune responses by cleaving mitochondrial antiviral signaling protein (MAVS) [7] and TIR domain-containing adaptor inducing interferon-β (TRIF) [8], two crucial adaptor molecules in the retinoic acid-inducible gene I (RIG-I) and Toll-like receptor 3 (TLR3) pathway, respectively. In addition, the NS3-4A protease has been reported to modulate epidermal growth factor receptor signaling by cleaving T-cell protein tyrosine phosphatase (TC-PTP) [9]. Finally, more recent studies have identified DNA damage-binding protein 1 (DDB1) [10], Riplet [11], complement component 4γ (C4γ) [12], glutathione peroxidase 8 (GPx8) [13], importin β1 [14] and Werner syndrome protein [15] as targets of the NS3-4A protease.

The identification of cellular substrates of the HCV NS3-4A protease should provide new insights into the HCV life cycle and the pathogenesis of hepatitis C. To this end, we have carried out a quantitative proteomics screen involving stable isotopic labeling using amino acids in cell culture (SILAC) coupled with mass spectrometry, resulting in the identification of the membrane-associated peroxidase GPx8 as a novel cellular substrate of the NS3-4A protease [13]. Here, we describe ovarian cancer immunoreactive antigen domain containing protein 1 (OCIAD1) as a further substrate identified by this approach.

OCIAD1, also designated as Asrij, is a relatively poorly characterized membrane protein believed to be involved in cancer development, neurodegenerative disease and stem cell homeostasis by integrating multiple signaling pathways such as Jak-STAT, Notch and phosphatidylinositol 3-kinase-Akt ([16] and [17] as well as refs. therein). We found that it is cleaved by the NS3-4A protease at Cys 38, close to a predicted transmembrane segment. Cleavage was observed in different experimental systems as well as in liver biopsies from patients with chronic hepatitis C. Proteolysis was found to be conserved across diverse members of the hepacivirus genus. Interestingly, OCIAD2, a homolog of OCIAD1 with a cysteine residue in a similar position and identical subcellular localization was not cleaved by NS3-4A, allowing us to study determinants for NS3-4A substrate selectivity by domain swapping experiments. Finally, OCIAD1 appears not to be involved in the viral life cycle *in vitro*, raising the possibility that NS3-4A-mediated cleavage of OCIAD1 may play a role in the pathogenesis of hepatitis C *in vivo*.

## Results

### Identification of OCIAD1 as a novel cellular substrate of the HCV NS3-4A protease

To identify new cellular substrates of the HCV NS3-4A protease, we have previously carried out two proteomics screens involving SILAC coupled with mass spectrometry [13]. In addition to GPx8, which has been described previously [13], these two independent screens performed in cells inducibly expressing NS3-4A have identified OCIAD1 as a potential cellular substrate of the viral protease. OCIAD1 was identified by 3 unique peptides in the first and 7 in the second screen. The main band for OCIAD1 was detected at an apparent molecular weight of 32.5 kDa, with a ratio (+*/*- tetracycline, i.e. in the absence *vs*. the presence of NS3-4A) of 2.61 and 2.77 in the two experiments. The cleaved form with a molecular weight of 29.5 kDa was detected only upon of NS3-4A expression in both screens.

**Fig 1:**
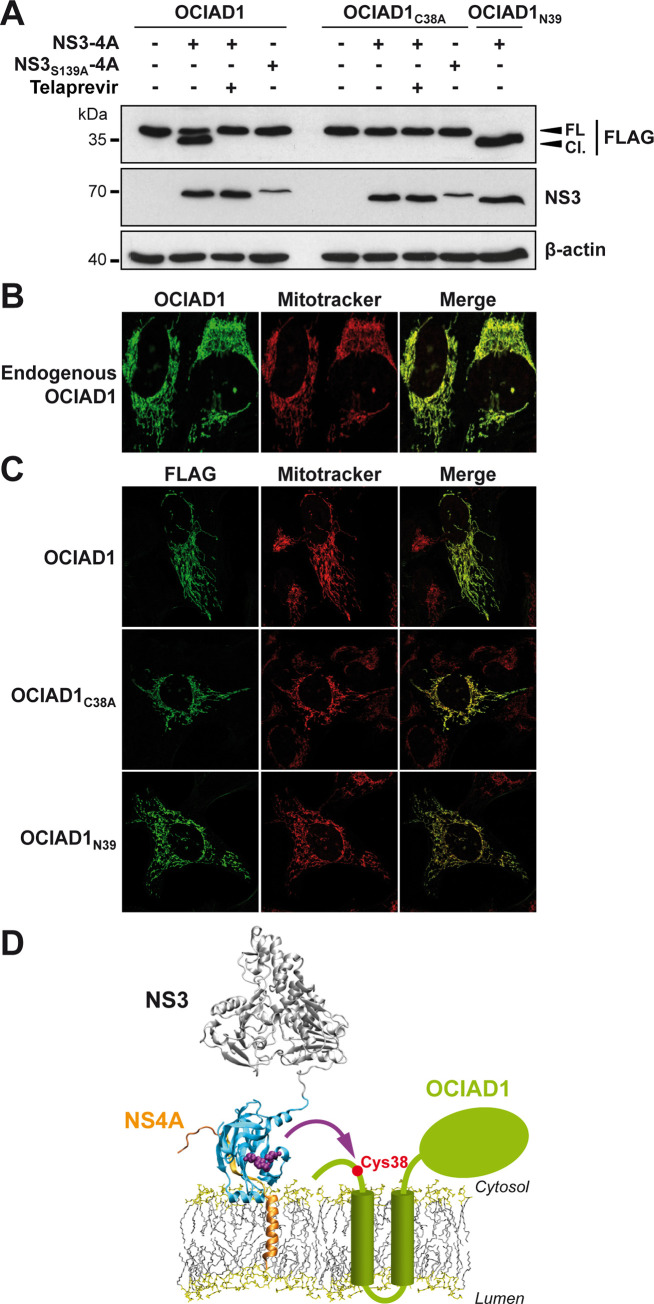
OCIAD1 is a novel cellular substrate of the HCV NS3-4A protease. **(A)** U-2 OS cells were transfected with the expression vectors pCMV-OCIAD1-FLAG, pCMV-OCIAD1_C38A_-FLAG or pCMV-OCIAD1_N39_-FLAG together with those encoding wild-type or proteolytically inactive (S139A) NS3-4A protease, as indicated. Twelve h post-transfection, cells were treated or not for 24 h with 2.5 μM telaprevir. Cell lysates were separated by 15% SDS-PAGE, followed by immunoblot with monoclonal antibodies anti-FLAG^®^M2 against the FLAG tag, 1B6 against NS3 or AC-15 against β-actin. Cl., cleaved OCIAD1; FL, full-length OCIAD1. **(B)** Endogenous OCIAD1 localizes to mitochondria. U-2 OS cells were subjected to immunofluorescence using a polyclonal antibody against OCIAD1 (green) and MitoTracker for staining of mitochondria (red). Mean Pearson’s coefficient was determined as 0.92 (n = 5). **(C)** NS3-4A–mediated cleavage does not alter the subcellular localization of OCIAD1. U-2 OS cells were transfected with plasmid pCMV-OCIAD1-FLAG (OCIAD1_wt_), pCMV-OCIAD1_C38A_-FLAG (OCIAD1_C38A_), or pCMV-OCIAD1_N39_-FLAG (OCIAD1_N39_). Thirty-six h post-transfection, cells were subjected to immunofluorescence using monoclonal antibody anti-FLAG^®^M2 against the FLAG tag (green) and MitoTracker (red). Mean Pearson’s coefficients were determined as 0.95, 0.85 and 0.88, respectively, for OCIAD1_wt_, OCIAD1C_38A_ and OCIAD1_N39_ (n = 5). **(D)** Tentative model of the cleavage of OCIAD1 by the NS3-4A protease. The model of NS3-4A on the membrane is from Brass V *et al*. (24). The NS3 serine protease domain is shown in cyan, with the catalytic triad in purple and NS4A in orange. OCIAD1 (green) harbors two predicted transmembrane segments and a C-terminal region oriented toward the cytosolic side. Cys 38 of OCIAD1 is highlighted by a red dot.

OCIAD1 is a 245-amino-acid (aa) protein of 35 kDa with two predicted transmembrane segments in the central region [18]. However, little is known about its function and no structure is available to date. To experimentally validate OCIAD1 as a substrate of NS3-4A, C-terminally FLAG-tagged OCIAD1 was co-expressed with proteolytically active NS3-4A or the inactive control NS3_S139A_-4A in U-2 OS cells. As shown in Fig 1A, OCIAD1 was cleaved by the active NS3-4A protease but not by the catalytically inactive control. To exclude artefacts of transfection and overexpression, we used telaprevir, a specific inhibitor of the NS3-4A protease, as additional control. Telaprevir did not alter the expression of NS3-4A but prevented the cleavage of OCIAD1 by the NS3-4A protease (Fig 1A).

Based on the migration pattern of the cleavage product of C-terminally FLAG-tagged OCIAD1 (3-4-kDa shift in apparent molecular weight), the cleavage was expected to occur close to the N terminus of the protein. Analysis of the primary sequence of OCIAD1 showed the presence of a cysteine at aa position 38. Since *trans*-cleavage by NS3-4A is known to occur after cysteine residues, we replaced this potential target residue by alanine (C38A). As shown in Fig 1A, OCIAD1_C38A_ was no longer cleaved by NS3-4A, indicating that the NS3-4A protease cleaves OCIAD1 at Cys 38. Moreover, an N-terminally truncated OCIAD1 construct, in which the first 38 amino acid residues were deleted (N39), comigrated with the cleaved product derived from full-length OCIAD1 (Fig 1A). Taken together, these results demonstrate that OCIAD1 is a novel cellular substrate of the HCV NS3-4A protease and that cleavage occurs at Cys 38.

OCIAD1 is a membrane protein that has been reported to localize to mitochondria and, to a minor extent, endosomes [19, 20]. Our immunofluorescence analyses confirmed that endogenous OCIAD1 localizes to mitochondria (Fig 1B). In addition, cleavage by the NS3-4A protease did not alter the subcellular localization of OCIAD1 (Fig 1C). In line with this observation, the uncleavable mutant OCIAD1_C38A_ and the N-terminally truncated construct OCIAD1_N39_ showed the same subcellular localization and colocalization with the mitochondrial marker (Fig 1C), indicating that OCIAD1 remains localized on mitochondria after NS3-4A-mediated cleavage. Human OCIAD1 is believed to possess two hydrophobic α-helices in aa regions 43–63 and 73–94 [20]. Hence, based on the position of the cleavage site in OCIAD1 as well as on the putative OCIAD1 topology, we propose in Fig 1D a tentative model of the cleavage of OCIAD1 by NS3-4A on the membrane.

### Endogenous OCIAD1 is cleaved in models of HCV infection and in liver biopsies from patients with chronic hepatitis C

**Fig 2:**
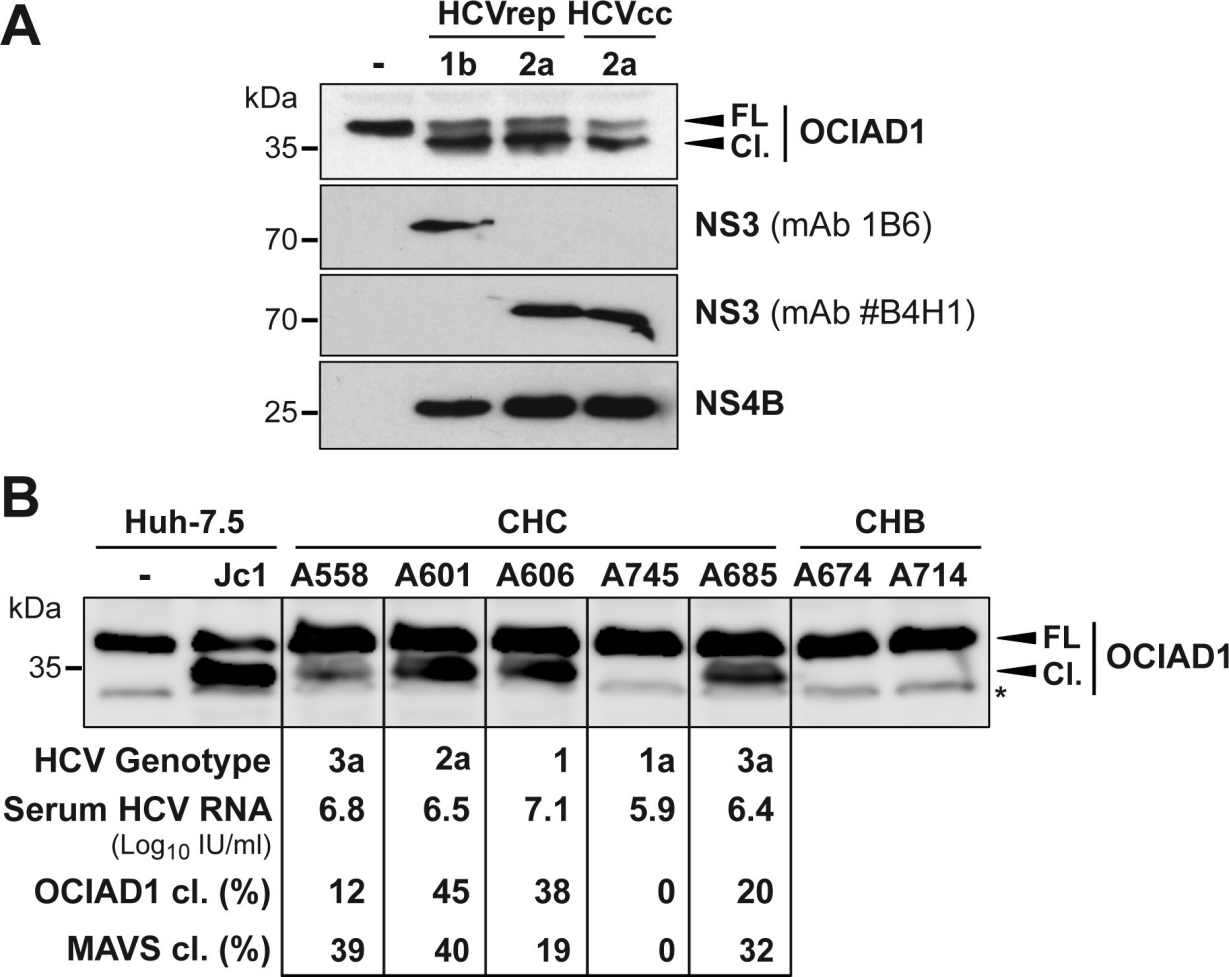
OCIAD1 is cleaved in model systems of HCV infection and in liver biopsies from patients with chronic hepatitis C. **(A)** Cleavage of OCIAD1 in HCV replicon and infection systems. Protein lysates obtained from naïve Huh-7.5 cells (-), cells replicating selectable subgenomic replicons (HCVrep) derived from the Con1 strain (genotype 1b) or the JFH1 strain (genotype 2a) as well as cells infected with cell culture-derived HCV (HCVcc; Jc1) were subjected to immunoblot using polyclonal antibodies against OCIAD1 or NS4B monoclonal antibodies 1B6 or #B4H1 against NS3 from genotypes 1b or 2a, respectively. **(B)** Cleavage of OCIAD1 in liver biopsies from patients with chronic hepatitis C. Protein lysates from liver biopsies of patients with chronic hepatitis C (CHC) or chronic hepatitis B (CHB), which served as control samples, were separated by 15% SDS-PAGE, followed by immunoblot with a polyclonal antibody against OCIAD1. A total of 20 μg protein per lane was loaded for the lysates from naïve or Jc1-infected Huh-7.5 cells while 100 μg protein per lane was loaded for the liver biopsy specimens. The asterisk indicates a nonrelevant background signal. The intensity of the signal corresponding to full-length (FL) and cleaved OCIAD1 (Cl.) were quantified using ImageJ software and served to calculate the percentage of OCIAD1 cleavage. The indicated percentage of mitochondrial antiviral signaling protein (MAVS) cleavage has been determined and reported in a previous study performed on the same samples [21].

To investigate whether OCIAD1 is cleaved by NS3-4A in models of HCV infection, we examined OCIAD1 in Huh-7.5 cells harboring subgenomic replicons derived from HCV genotype 1b (Con1) and 2a (JFH1) as well as in Huh-7.5 cells infected with cell culture-derived HCV (HCVcc; Jc1 virus). As shown in Fig 2A, cleavage of endogenous OCIAD1 was observed in Huh-7.5 cells harboring subgenomic Con1 and JFH1 replicons as well as in cells infected with Jc1 HCVcc. Importantly, we also found cleavage of OCIAD1 in liver biopsies from patients with chronic hepatitis C. Liver biopsy samples from 5 patients with chronic hepatitis C and two patients with chronic hepatitis B as controls were analyzed by immunoblot using a monoclonal antibody (mAb) against OCIAD1. Both full-length and cleaved forms of OCIAD1 were detected in liver biopsy specimens from patients with chronic hepatitis C whereas only the full-length protein was detected in the controls (Fig 2B). The percentage of OCIAD1 cleavage varied widely among the 5 patients with chronic hepatitis C, ranging from 0 to 45% and correlating roughly with the serum viral loads as well as the percentage of MAVS cleavage previously observed in the same biopsy specimens [21].

Taken together, our data show that OCIAD1 is cleaved not only in heterologous expression systems but also in models of HCV infection as well as in the liver of patients with chronic hepatitis C. Moreover, our observations demonstrate that OCIAD1 is cleaved by NS3-4A derived from different HCV genotypes (1a, 1b, 2a, and 3a) represented by the Con1 and JFH1 cell culture adapted clones and the viruses circulating in the sera of patients included in this study.

### Human OCIAD1 is susceptible to NS3-4A proteases from diverse members of the hepacivirus genus

**Fig 3:**
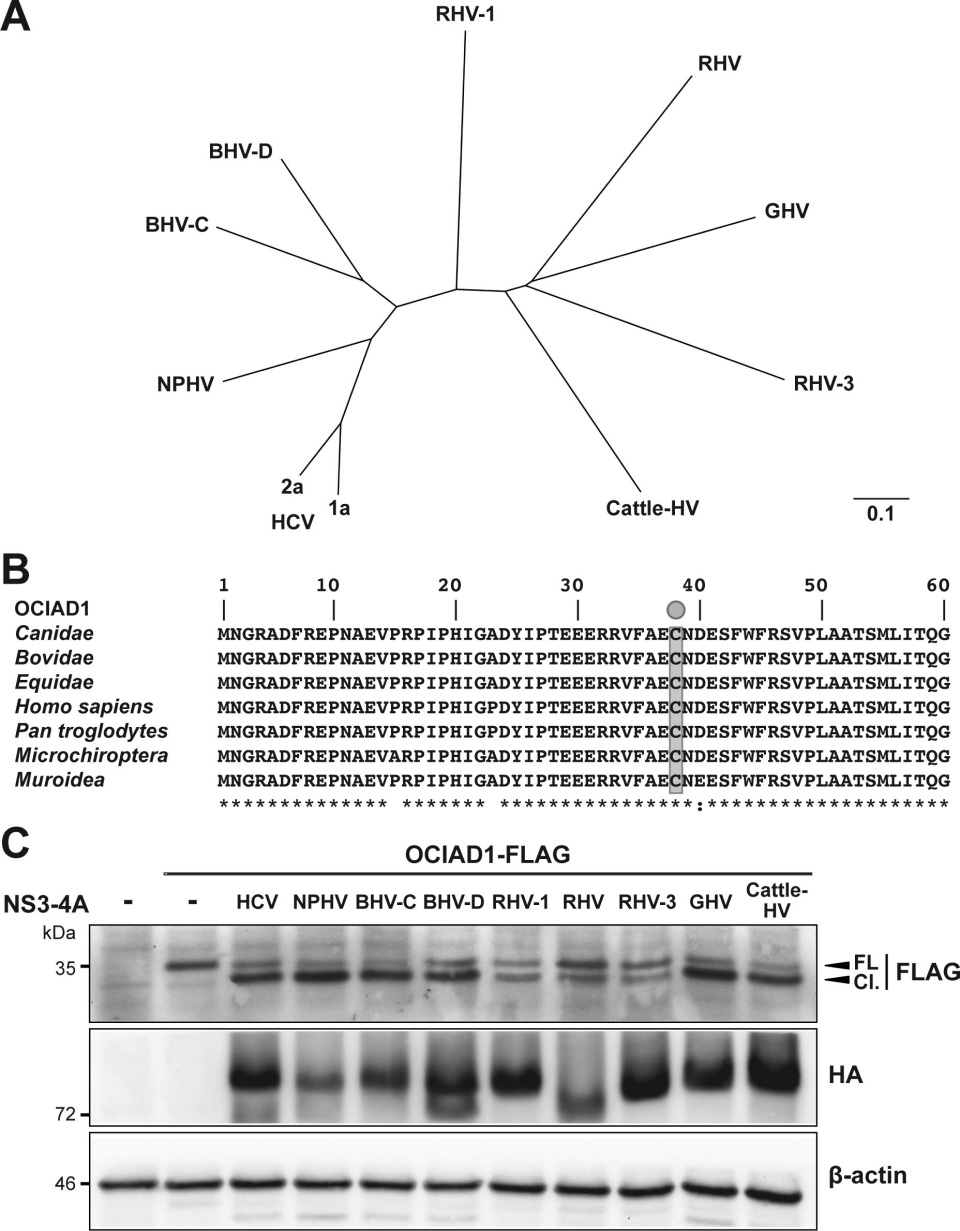
OCIAD1 is a conserved cleavage target of hepaciviral NS3-4A proteases. **(A)** Phylogenetic tree deduced from the NS3-4A protein sequences of different hepaciviruses. NS3-4A protein sequences from hepatitis C virus (HCV) genotypes 1a and 2a (NP_671491 and BAB32872, respectively), nonprimate hepacivirus (NPHV; JQ4340019), bat hepaciviruses (BHV-C and BHV-D; KC796090 and KC796074, respectively), rodent hepaciviruses (RHV, RHV-1 and RHV-3; GenBank accession numbers NC_021153, KC411777 and KC411807, respectively), guereza hepacivirus (GHV; KC551800) and cattle hepacivirus (Cattle-HV; NC_026797) retrieved from GenBank were aligned with ClustalW, followed by phylogenetic tree building using the neighbor-joining method (Geneious 11.1.5; Biomatters, Auckland, New Zealand). The scale bar indicates the number of amino acid substitutions per site. **(B)** Alignment of OCIAD1 target sequences from the hosts of the different hepaciviruses. OCIAD1 sequences from Canidae (*Canis lupus familiaris*; GenBank accession number XP_005628232), Equidae (*Equus caballus*; XP_005608844), human (*Homo sapiens*; NP_001014446) and chimpanzee (*Pan troglodytes*; XP_001151365), Bovidae (*Bos taurus*; Q5E948), Microchiroptera (*Eptesicus fuscus*; XP_008138514) and Muroidea (*Peromyscus maniculatus bairdii*; XP_006979757) were retrieved from GenBank and aligned using ClustalW. The NS3-4A target residue Cys 38 is highlighted in grey. The degree of physicochemical amino acid conservation at each position can be inferred from the similarity index according to ClustalW convention (asterisk, invariant; colon, highly similar; dot, similar) [23]. **(C)** Effect of hepaciviral NS3-4A proteases on human OCIAD1. pCMV-OCIAD1-FLAG was cotransfected with plasmids allowing the expression of hemagglutinin (HA)-tagged NS3-4A proteases from the different hepaciviruses. Protein lysates harvested 48 h post-transfection were subjected to immunoblot using monoclonal antibodies anti-FLAG^®^M2 against the FLAG tag, 16B12 against the HA tag or AC-15 against β-actin.

Cleavage of human MAVS by NS3-4A protease is a conserved innate immune evasion strategy adopted by diverse hepaciviruses [22] which may contribute toward a zoonotic transmission potential. The primary sequences of the NS3-4A region from the viral polyproteins of HCV (Con1 and JFH1 strains), non-primate hepacivirus, bat hepaciviruses, rodent hepaciviruses, guereza hepacivirus and cattle hepacivirus show strong divergence, with identities ranging from 34.8 to 58.8% (Fig 3A). Of note, the OCIAD1 aa sequence of the natural hosts of the different hepaciviruses showed a high degree of identity with the human ortholog, ranging from 84.6 to 97.6%, especially in the N-terminal region where Cys 38 and the preceding residues are conserved (Fig 3B). Here, we explored whether the orthologous proteases cleave human OCIAD1 at the identified cleavage site. To this end, human OCIAD1 and the proteases of the different hepacivirus strains were co-expressed in 293T cells and protein lysates analyzed by immunoblot. Our results revealed that OCIAD1 is susceptible to cleavage by all hepaciviral NS3-4A proteases, although a somewhat less efficient cleavage was observed with the rodent hepaciviral proteases (Fig 3C). Our results suggest that cleavage of OCIAD1 is an important evolutionary conserved attribute of all hepaciviral infections in their cognate hosts.

### OCIAD2 is not cleaved by the HCV NS3-4A protease

**Fig 4:**
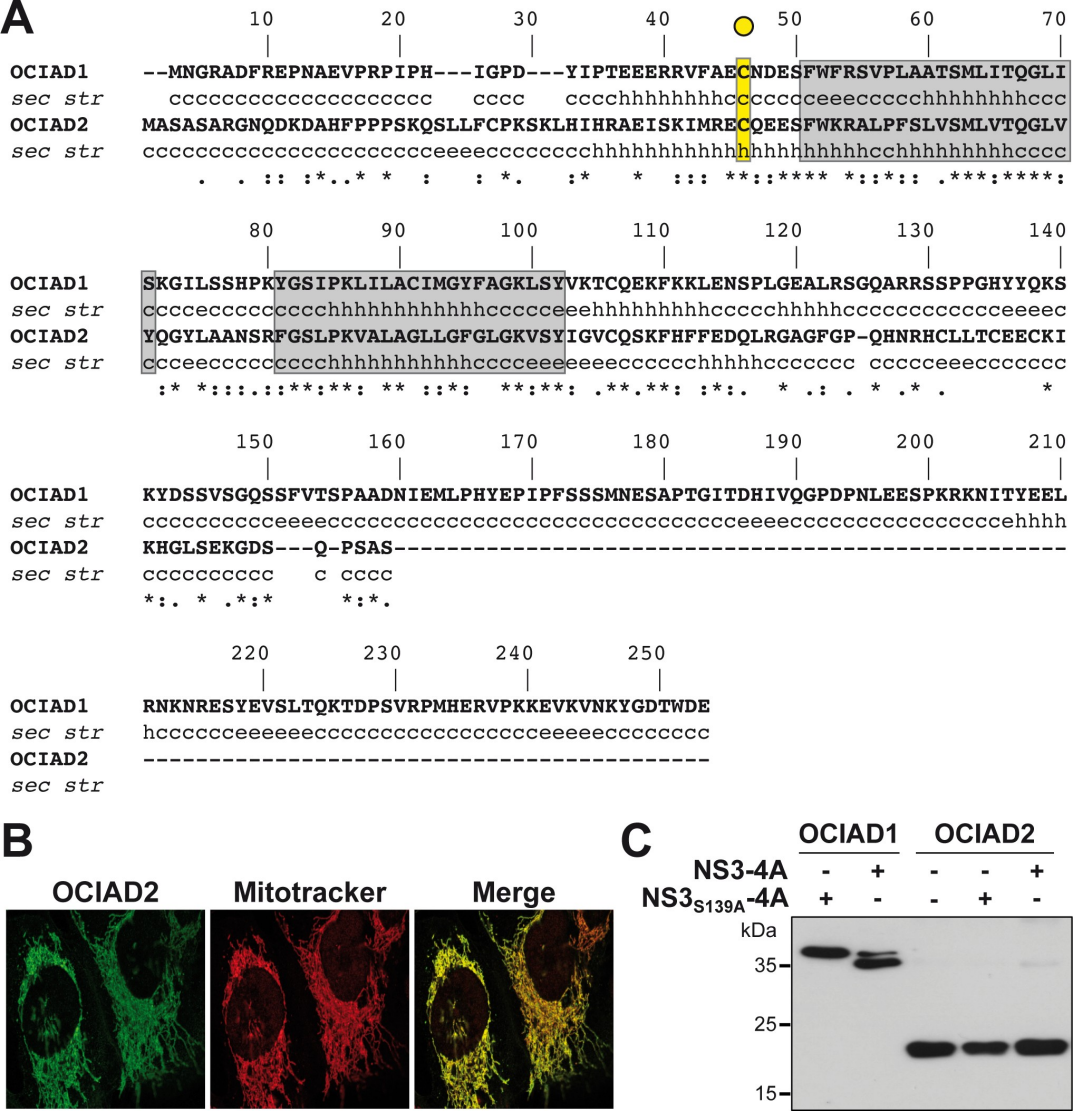
OCIAD2 is not cleaved by the HCV NS3-4A protease. **(A)** Sequence alignment of human OCIAD1 and OCIAD2. The degree of physicochemical amino acid conservation at each position can be inferred from the similarity index according to ClustalW convention (asterisk, invariant; colon, highly similar; dot, similar) [23]. The NS3-4A target residue of OCIAD1 (Cys 38) is highlighted in yellow. Grey boxes denote the transmembrane segments predicted by TMpred. **(B)** Endogenous OCIAD2 localizes to mitochondria. U-2 OS cells were subjected to immunofluorescence using a polyclonal antibody against OCIAD2 (green) and MitoTracker for staining of mitochondria (red). **(C)** OCIAD2 is not cleaved by NS3-4A. pCMV-OCIAD1-FLAG and pCMV-OCIAD2-FLAG constructs were co-transfected together with active or inactive HCV NS3-4A protease, and protein lysates were analyzed after separation on 15% SDS-PAGE by immunoblotting using monoclonal antibody anti-FLAG^®^M2.

While little is known about the function of OCIAD1, a homolog designated as OCIAD2 has been identified as a modulator of γ-secretase, an enzyme that stimulates the production of amyloid β in the early stage of Alzheimer’s disease [24]. It displays 40% aa sequence identity with OCIAD1 in the OCIA domain extending from aa 23 to 108, i.e. the region surrounding the NS3-4A cleavage site as well as the predicted transmembrane segments (Fig 4A) and shares the same subcellular localization at mitochondria (Fig 4B) [20]. While sharing similar features with OCIAD1, OCIAD2 is not a substrate of the NS3-4A protease, as revealed by co-expression experiments (Fig 4C). In line with these observations, OCIAD2 was detected in our mass spectrometry data but did not show any evidence of NS3-4A-induced cleavage.

### The amino acid sequence surrounding the cleavage site as well as the predicted transmembrane segments contribute to substrate selectivity

**Fig 5:**
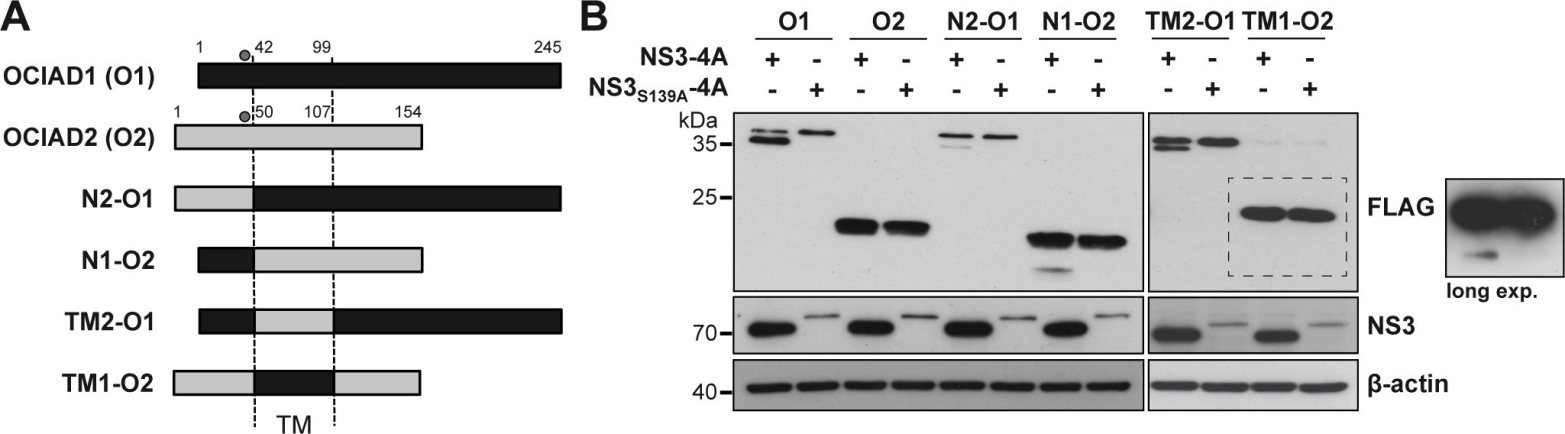
Cleavage of OCIAD1-OCIAD2 chimeras. **(A)** Schematic representation of the OCIAD1-OCIAD2 chimeras. The dots indicate the position of the conserved cysteine target residue. TM, predicted transmembrane region. **(B)** Chimeras were co-expressed with active or inactive HCV NS3-4A protease, followed by separation on 15% SDS-PAGE and immunoblot using monoclonal antibodies anti-FLAG^®^M2, 1B6 or AC-15 against the FLAG tag, NS3 and β-actin, respectively.

Based on the common features of OCIAD1 and OCIAD2 such as mitochondrial localization, a conserved cysteine residue and two predicted transmembrane segments, we prepared a panel of chimeric constructs by swapping the N-terminal regions and predicted transmembrane segments of OCIAD1 and OCIAD2 (Fig 5A).

As shown in Fig 5B, and consistent with our previous results, OCIAD1 was cleaved by the active NS3-4A protease but not by the inactive control whereas OCIAD2 was not cleaved by NS3-4A. Interestingly, swapping of the N-terminal regions resulted in cleavage of both proteins, albeit to a much lower extent as compared to wild-type OCIAD1 (Fig 5, constructs N2-O1 and N1-O2). Densitometry analyses revealed about 10% cleavage as opposed to 75% for wild-type OCIAD1. Hence, the N-terminal region of OCIAD1 confers to OCIAD2 some degree of susceptibility to NS3-4A-mediated cleavage. However, additional determinants for selectivity appear to be involved, as maximal cleavage efficiency was not reached. Furthermore, cleavage of the N2-O1 chimera indicates that determinants other than the sequence surrounding the cleavage site may contribute to the substrate selectivity of NS3-4A.

To investigate the role of the determinants for membrane association of OCIAD1 in NS3-4A protease substrate selectivity, we prepared chimeric constructs by swapping the transmembrane regions of OCIAD1 and OCIAD2, i.e. TM2-O1 and TM1-O2. As shown in Fig 5B, our results indicate that the OCIAD1 chimera harboring the predicted transmembrane segments of OCIAD2 was cleaved by NS3-4A, albeit with reduced efficacy (about 40% cleavage as opposed to 75% for wild-type OCIAD1). A very minor proportion of cleaved product, revealed after long exposure of the immunoblot, was observed for the TM1-O2 chimera, suggesting that the predicted transmembrane segments themselves may contribute to the substrate selectivity of NS3-4A.

Taken together, our data underline the importance of the sequence surrounding the cleavage site and suggest that the transmembrane domain may contribute to the substrate selectivity of NS3-4A.

### OCIAD1 does not affect the HCV life cycle *in vitro*

To examine the functional significance of OCIAD1 and its cleavage in the HCV life cycle, siRNA-mediated silencing as well as overexpression and rescue experiments with siRNA-resistant versions of OCIAD1 were carried out. Huh-7.5 cells were transfected with OCIAD1-targeting siRNAs #1 or #2 or a non-targeting control, followed by transduction with recombinant retroviruses allowing the expression of siRNA-resistant versions of OCIAD1, the uncleavable mutant OCIAD1_C38A_, the N-terminally truncated construct OCIAD1_N39_ or the green fluorescent protein (GFP) as a control, and, finally, infection with HCVcc.

**Fig 6:**
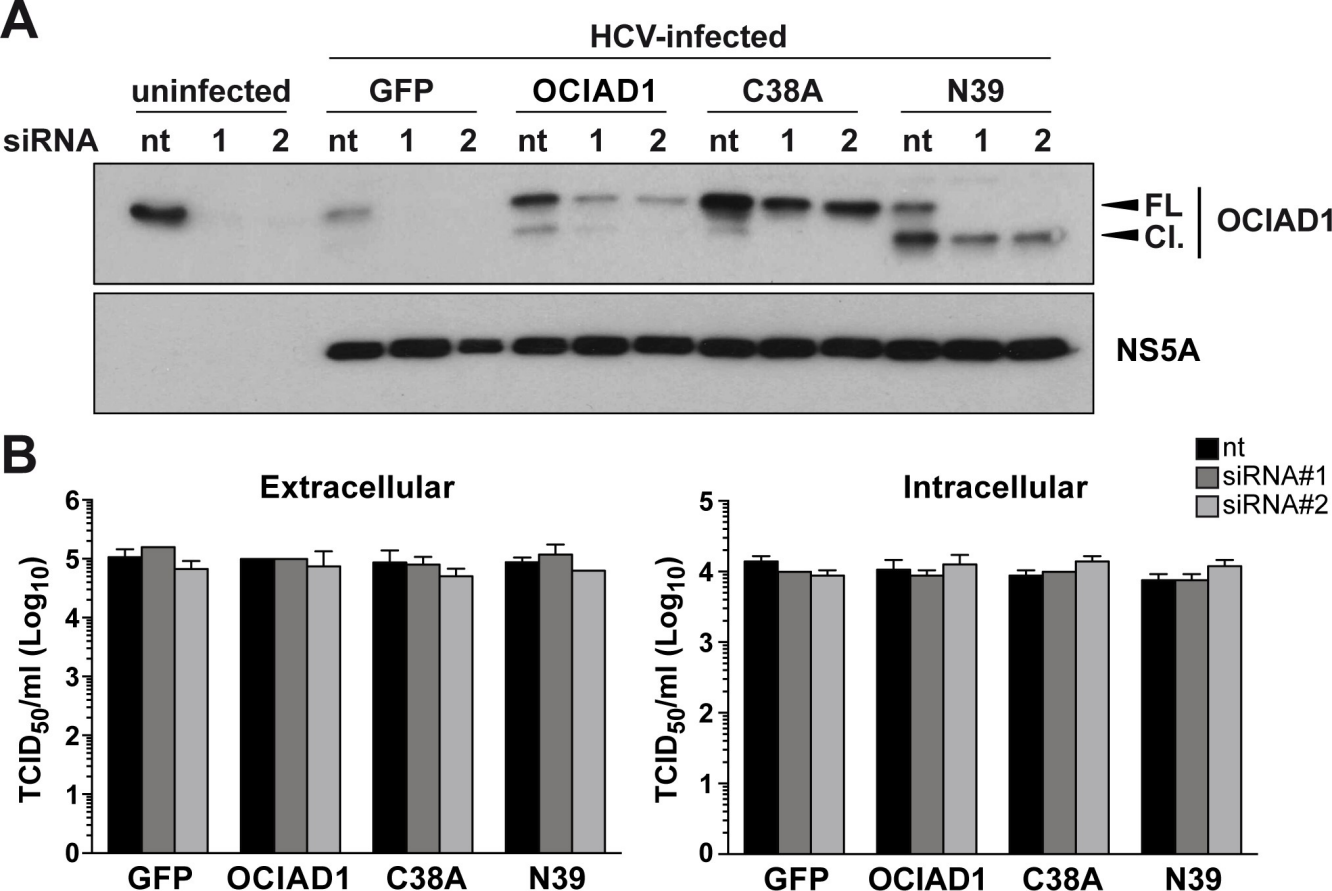
OCIAD1 is not involved in the HCV life cycle *in vitro*. **(A)** Huh-7.5 cells were transfected with OCIAD1 siRNAs (#1, #2) or non-targeted control siRNA (nt) 24 h prior to transient transduction with recombinant retroviruses (multiplicity of infection [MOI] = 10) expressing either wild-type OCIAD1 (OCIAD1), uncleavable mutant C38A (C38A), truncated mutant N39 (N39) or the green fluorescent protein (GFP). Twenty-four h post-transduction, the cells were inoculated with cell culture-derived HCV (Jc1) at a MOI of 0.5 for 4 h. Cells were harvested 72 h post-infection and protein lysates were separated by 12% SDS-PAGE, followed by immunoblot with a polyclonal antibody against OCIAD1 or monoclonal antibody 9E10 against HCV NS5A. **(B)** Extra- and intracellular infectivities were determined 72 h post-infection by 50% tissue culture infective dose (TCID_50_) determination. Each histogram represents the mean ± SEM of two independent experiments performed in triplicate.

As shown in Fig 6A, endogenous OCIAD1 was silenced efficiently by both OCIAD1 siRNAs #1 and #2. OCIAD1 could be rescued by the expression of siRNA-resistant OCIAD1 as well as OCIAD1_C38A_ and OCIAD1_N39_ to levels similar to the endogenous protein. Silencing of OCIAD1 had no effect on intra- and extracellular infectivities of HCVcc (Fig 6B), indicating that OCIAD1 is required neither for viral replication nor infectious virus production. Moreover, cells expressing siRNA-resistant OCIAD1 as well as OCIAD1_C38A_ and OCIAD1_N39_ yielded similar titers of intra- and extracellular infectious virus as compared to cells expressing GFP. These results indicate that OCIAD1 and its cleavage by NS3-4A do not affect the HCV life cycle *in vitro*.

## Discussion

Here, we describe OCIAD1 as host mitochondrial substrate of the HCV NS3-4A protease. Cleavage occurs at Cys 38 and was observed in different experimental systems, including heterologous expression, the replicon and HCVcc systems, as well as in liver biopsies from patients with chronic hepatitis C. NS3-4A from different HCV genotypes as well as a range of hepacivirus species efficiently cleaved OCIAD1. Domain swapping with OCIAD2, a homolog of OCIAD1 which is not cleaved by NS3-4A, indicates that the sequence surrounding the cleavage site as well as the predicted transmembrane segments contribute to substrate selectivity. Finally, OCIAD1 did not affect the HCV life cycle *in vitro*, raising the possibility that OCIAD1 and its cleavage may play a role in the pathogenesis of hepatitis C *in vivo*.

The consensus cleavage site of NS3-4A in the HCV polyprotein is represented by the sequence D/E-X-X-X-X-C/T↓S/A-X-X-X (where X represents any amino acid) ([6] and references therein). However, many cellular proteins display the consensus cleavage sequence and yet only very few are cleaved by NS3-4A. Moreover, the cellular substrates identified so far, including OCIAD1, have less canonical cleavage sites. Quite strikingly, OCIAD1 has an arginine, i.e. a basic residue, in the P6 position which is usually occupied by an acidic residue. In addition, it has an aspartate in the P1' position which is usually occupied by a residue with a small side chain. Hence, substrate specificity of NS3-4A appears to be conferred by additional mechanisms.

Analyses of aa sequences and structures of MAVS, GPx8, and OCIAD1, which are transmembrane proteins and cellular substrates of NS3-4A, show that their cleavage sites are located a few aa away from their transmembrane segments, raising the possibility that positioning of the cleavage site with respect to the membrane may contribute to substrate selectivity of the NS3-4A protease. Indeed, the nuclear magnetic resonance structure of the transmembrane segment of NS4A and the deduced model of full-length NS3-4A revealed that the active site of the protease is positioned in close proximity to the membrane [25]. Therefore, substrate cleavage likely has to satisfy these topological constraints. The model proposed in Fig 1D fits very well with both the topology of the NS3-4A protease active site and the cleavage site. Interestingly, NS3-4A-mediated cleavage can occur at different cellular membrane compartments, including the endoplasmic reticulum (GPx8) [13], mitochondria or mitochondria-associated membranes (MAVS and OCIAD1) [26, 27] (this study) and peroxisomes (MAVS) [28]. However, other cellular substrates such as TRIF, DDB1, TC-PTP, Riplet and C4γ are not considered as being membrane proteins [8–12] but they may be part of a protein complex and thereby become accessible to the catalytic site of the NS3-4A protease in an appropriate topology.

OCIAD2, a homolog of OCIAD1, has similar subcellular localization and a cysteine residue at the position corresponding to the cleavage site of OCIAD1. Despite the similarities with OCIAD1, OCIAD2 is not cleaved by HCV NS3-4A, as revealed by the absence of cleavage by the viral protease in co-expression settings (Fig 4C) as well as in our SILAC screens. Taking advantage of the homology of these two proteins and of their different susceptibility to cleavage by the NS3-4A protease, we prepared chimeric constructs where different domains were swapped, including the N-terminal region and the transmembrane region. Swapping of the N-terminal regions of OCIAD1 and OCIAD2, comprising the cleavage site, led to partial cleavage of the N2-OCIAD1 and reduced cleavage of the N1-OCIAD2 chimera, indicating that the sequence surrounding the potential cleavage sites may be one of the factors determining the substrate selectivity of NS3-4A. Secondary structure predictions using a panel of algorithms including DSC, MLRC and PHD (available at https://npsa-prabi.ibcp.fr) suggests that Cys 46 of OCIAD2 (corresponding to Cys 38 of OCIAD1) may reside in an extended α-helix, whereas the cleavage site of OCIAD1 is predicted to reside in an unstructured segment. The secondary structure of a protein segment is likely an important determinant of the substrate selectivity of NS3-4A. Indeed, peptide bonds within α-helices are poorly accessible to proteases because of the steric hindrance imposed by the framework of hydrogen bonds that stabilize the helix ([29] and references therein). Of note, the secondary structure of the cleavage site of MAVS and GPx8, two well-characterized cellular substrates of NS3-4A, are predicted to be unstructured by the same algorithms. Thus, one hypothesis explaining the substrate selectivity of NS3-4A may reside in the folding and secondary structure of the target protein sequence.

Several cellular substrates of the NS3-4A protease, including MAVS, GPx8 and TC-PTP, possess a transmembrane region which likely favorably positions the substrates for cleavage by HCV NS3-4A. To address whether the predicted transmembrane region of OCIAD1 plays a role in the substrate selectivity, OCIAD1 and OCIAD2 constructs with swapped transmembrane segments were prepared. Of note, the transmembrane region is the region showing the highest degree of conservation between the two homologous proteins, i.e. 24/52 identical residues (Fig 4A). Swapping of the segments resulted in very limited cleavage of the TM1-OCIAD2 chimera and significantly reduced cleavage of the TM2-OCIAD1 chimera, suggesting a role of the transmembrane segments in the substrate selectivity of NS3-4A. The contribution to the substrate recognition may be explained by a conformational change of the cleavage site and/or by an interaction with transmembrane segments of other proteins, including the protease itself. The NS4A transmembrane α-helix displays numerous well-conserved small aa residues, notably on one side of the helix [25]. Mutants harboring single conserved amino acid substitutions have been well characterized in terms of polyprotein processing, membrane association, replication complex assembly and RNA replication [25]. However, these substitutions did not abrogate the cleavage of OCIAD1 or of other cellular substrates such as MAVS. Among the single aa substitutions tested, mutation G8L has been shown to prevent dimerization of the NS4A transmembrane segment [30]. Therefore, our results suggest that dimerization of NS4A may be not necessary for NS3-4A-mediated cleavage of OCIAD1 and MAVS. Future studies, including more extensive mutagenesis, may provide further insights into the contribution of the transmembrane domain of OCIAD1 to NS3-4A protease substrate selectivity.

To investigate the role of OCIAD1 and its cleavage in the HCV life cycle, knockdown as well as overexpression and rescue experiments with siRNA-resistant versions of OCIAD1 were carried out in Huh-7.5 cells infected with HCVcc (Fig 6). Similar, results were obtained after transfection of genotype 1b (Con1) and genotype 2a (JFH1) RNA replicons. Our results did not reveal any impact of OCIAD1 or its cleavage on the entire viral life cycle in the HCVcc system *in vitro*, raising the possibility that OCIAD1 may be involved in the pathogenesis of hepatitis C *in vivo*.

Little is known about the function of OCIAD1 and a structure is not available to date. OCIAD1 was first identified by immunoscreening of an ovarian carcinoma cDNA expression library with ascites fluid from patients with ovarian cancer [18]. It has subsequently been shown to be upregulated in metastatic as compared to primary ovarian cancer tissue [16] as well as in other cancer types. In addition to cancer development, OCIAD1 has been involved in neurodegenerative disease and stem cell homeostasis by integrating multiple signaling pathways such as Jak-STAT, Notch and phosphatidylinositol 3-kinase-Akt ([16] and [17] as well as refs. therein). Hence, investigating a potential role of OCIAD1 and its cleavage in the pathogenesis of hepatitis C will require experimental models which are still limited to date.

In conclusion, OCIAD1 represents a novel cellular substrate of the HCV NS3-4A protease. It does not appear to be involved in the viral life cycle *in vitro* but may play a role in the pathogenesis of hepatitis C *in vivo*, which shall be explored in future studies.

## Materials and methods

### Cell lines and reagents

U-2 OS human osteosarcoma, 293T human embryonic kidney and Huh-7.5 human hepatocellular carcinoma [31] (kindly provided by Charles M. Rice, The Rockefeller University, New York, NY) cell lines were maintained in Dulbecco’s modified Eagle medium supplemented with 10% fetal bovine serum. X-tremeGENE HP DNA Transfection Reagent (Roche, Basel, Switzerland) was used for transient transfection according to the manufacturer's instructions. Telaprevir was kindly provided by Johan Neyts (Rega Institute for Medical Research, Leuven, Belgium).

MAb #337 against HCV NS3 was raised by immunization of mice with a recombinant NS3 protein derived from the JFH1 isolate. Hybridomas were established according to standard protocols and hybridoma supernatants were screened using an ELISA based on the recombinant protein used for immunization. Reactive hybridoma clones were subcloned and clone #337 was chosen. MAb 1B6 against HCV NS3 has been described [32]. Polyclonal antibody #86 against NS4B [33] was kindly provided by Ralf Bartenschlager (University of Heidelberg, Germany). Polyclonal antibodies against OCIAD1 and OCIAD2 were from Proteintech (Rosemont, IL) and (Sigma-Aldrich St. Louis, MO), respectively. MAbs anti-FLAG^®^M2 and AC-15 against the FLAG tag and β-actin, respectively, were from Sigma-Aldrich. MAb 16B12 against the hemagglutinin tag was obtained from BioLegends (San Diego, CA). Secondary antibodies were HRP-conjugated anti-mouse (GE Healthcare, Buckinghamshire, UK) and anti-rabbit (Agilent Dako, Santa Clara, CA) IgG antibodies.

### Plasmids

Primers used in this study are listed in Table 1.

**Table 1:** List of primers used in this study.

Name	Nucleotide sequence (5' → 3')
OCIAD1-fd	GGTCACGGATGCGTGTGGGG
OCIAD1-rv	TGCTGAGCTCAAAGCATGCAGGT
OCIAD1-Bsp-fd	ATGATG**TCCGGA**AATGGGAGGGCTGATTTTCG
OCIAD1-Bam-rv	ATGATG**GGATCC**CTCATCCCAAGTATCTCCAT
OCIAD2-fd	CTGGGCTTGGCAACGAGGGAC
OCIAD2-rv	AACTTCGAAAGTCACAGACACAGA
OCIAD2-Bsp-fd	ATGATG**TCCGGA**GCTTCAGCGTCTGCTCGT
OCIAD2-Bam-rv	ATGATG**GGATCC**GGAAGCTGAAGGCTGAGAGTC
OCIAD1-C38A-fd	AGAGTCTTCGCAGAAGCCAATGATGAAAGCTTCTG
OCIAD1-C38A-rv	GAAGCTTTCATCATTGGCTTCTGCGAAGACTCTCC
OCIAD1-N39-Bsp-fd	ATGATG**TCCGGA**AATGATGAAAGCTTCTGG
OCIAD1-Eco-fd	AGTAGT**GAATTC**ACCATGAATGGGAGGGCTGAT
OCIAD1-Not-rv	AGTAGT**GCGGCCGC**TCACTCATCCCAAGTATC
OCIAD1_N39-Eco-fd	AGTAGT**GAATTC**ACCATGAATGATGAAAGCTTCTGG
OCIAD1-siRNA-1fd	TTGGCTGCGACGTCCATGCTCATCACTCAAGGATTAATTAGT
OCIAD1-siRNA-1rv	TCCTTGAGTGATGAGCATGGACGTCGCAGCCAAAGGCACAGATCT
OCIAD1-siRNA-2fd	CCCCAGCGGCGGATAATATCGAGATGCTTCCTCATTATGAG
OCIAD1-siRNA-2rv	AGGAAGCATCTCGATATTATCCGCCGCTGGGGATGTCACAAAAGA
OCIAD1-siRNA-3fd	AAATATGGCAGTATTCCGAAGTTGATACTTGCTTGTATCATG
OCIAD1-siRNA-3rv	AGCAAGTATCAACTTCGGAATACTGCCATATTTGGGATGACTTGA
N_2-OCIAD1-fd	ATGCGAGAATGTCAGGAAGAAAGCTTCTGGTTCAGATCTGTG
N_2-OCIAD1-rv	CACAGATCTGAACCAGAAGCTTTCTTCCTGACATTCTCGCAT
N_1-OCIAD2-fd	CTTCGCAGAATGCAATGATGAAAGTTTCTGGAAGAGAGCTCTG
N_1-OCIAD2-rv	CAGAGCTCTCTTCCAGAAACTTTCATCATTGCATTCTGCGAAG
TM_2-OCIAD1-fd1	CTTCGCAGAATGCAATGATGAAAGTTTCTGGAAGAGAGCTCTG
TM_2-OCIAD1-rv1	CAGAGCTCTCTTCCAGAAACTTTCATCATTGCATTCTGCGAAG
TM_2-OCIAD1-fd2	GTATCATACATAGGAGTATGCCAAGAGAAATTCAAGAAACTTG
TM_2-OCIAD1-rv2	CAAGTTTCTTGAATTTCTCTTGGCATACTCCTATGTATGATAC
TM_1-OCIAD2-fd1	ATGCGAGAATGTCAGGAAGAAAGCTTCTGGTTCAGATCTGTG
TM_1-OCIAD2-rv1	CACAGATCTGAACCAGAAGCTTTCTTCCTGACATTCTCGCAT
TM_1-OCIAD2-fd2	CTTTCTTATGTGAAAACTTGCCAGAGTAAATTCCATTTTTTTG
TM_1-OCIAD2-rv2	CAAAAAAATGGAATTTACTCTGGCAAGTTTTCACATAAGAAAG

Restriction sites are in bold.

cDNA was prepared by oligo-dT priming from total cellular RNA isolated from U-2 OS cells, followed by PCR using specific primers OCIAD1-fd and OCIAD1-rv or OCIAD2-fd and OCIAD2-rv to amplify OCIAD1 and OCIAD2 cDNAs, respectively. OCIAD1 and OCIAD2 coding sequences were then PCR amplified by OCIAD1-BspEI-fd or OCIAD2-BspEI-fd and OCIAD1-BamHI-rv or OCIAD2-BamHI-rv, followed by *Bam*HI/*Bsp*EI digestion and cloning into the pcDNA3.1(+)-derived plasmid pCMV-X-FLAG described previously [13], allowing fusion of a FLAG tag at the C terminus of a given protein, yielding plasmids pCMV-OCIAD1-FLAG and pCMV-OCIAD2-FLAG, respectively. Cys 38 in OCIAD1 was replaced by alanine (C38A) by using the QuickChange^TM^ Site-Directed Mutagenesis Kit (Stratagene, La Jolla, CA) as well as primers OCIAD1-C38A-fd and OCIAD1-C38A-rv, yielding pCMV-OCIAD1_C38A_-FLAG. In addition, an OCIAD1 mutant truncated of the first 38 aa was prepared by PCR amplification using primers OCIAD1-N39-BspEI-fd and OCIAD1-BamHI-rv, followed by *Bsp*EI/*Bam*HI digestion and cloning into pCMV-X-FLAG, yielding pCMV-OCIAD1_N39_-FLAG.

siRNA-resistant OCIAD1 expression constructs were prepared by site-directed mutagenesis, as described above, using pCMV-OCIAD1-FLAG or pCMV-OCIAD1_C38A_-FLAG as template and, in three successive steps, primer pairs OCIAD1-siRNA-1fd/OCIAD1-siRNA-1rv, OCIAD1-siRNA-2fd/OCIAD1-siRNA-2rv and OCIAD1-siRNA-3fd/OCIAD1-siRNA-3rv. The constructs harboring the three mutated sites served as templates for PCR amplification with forward primers OCIAD1-EcoRI-fd or OCIAD1_N39-EcoRI-fd and reverse primer OCIAD1-Not-rv, followed by cloning into pLPCX (Clontech, Mountain View, CA), yielding retroviral constructs pLPCX-OCIAD1wt, pLPCX-OCIAD1_C38A_ and pLPCX-OCIAD1_N39_.

Plasmids pCMVNS3-4A and pCMVNS3_S139A_-4A, the latter harboring an inactivating Ser-to-Ala mutation in the NS3-4A protease active site, have been described [25, 32].

HCV JFH1- and Con1-derived subgenomic replicon constructs harboring a neomycin resistance reporter gene, pFKi389NeoNS3-3’_δg_JFH [34] and pCon1/SG-Neo(I)/AflII [35], respectively, as well as the full-length HCV Jc1 construct pFK-JFH1/J6/C-846_δg [36] were kindly provided by Ralf Bartenschlager (University of Heidelberg, Germany) and Charles M. Rice.

Lentiviral pWPI plasmids expressing the hepaciviral NS3-4A variants with an N-terminal hemagglutinin tag have been described [22].

OCIAD1-OCIAD2 chimeras were prepared using pCMV-OCIAD1-FLAG or pCMV-OCIAD2-FLAG as backbone. Briefly, the constructs were generated by two-step overlap extension PCR using OCIAD1-BspEI-fd or OCIAD2-BspEI-fd with either N_1-OCIAD2-rv or N_2-OCIAD1-rv and N_1-OCIAD2-fd or N_2-OCIAD1-fd with OCIAD2-BamHI-rv or OCIAD1-BamHI-rv, followed by full-length amplification with forward primer OCIAD1-BspEI-fd or OCIAD2-BspEI-fd and reverse primer OCIAD2-BamHI-rv or OCIAD1-BamHI-rv, yielding pCMV-N_1-OCIAD2-FLAG and pCMV-N_2-OCIAD1-FLAG, respectively. Similarly, to generate the TM2-O1 and TM1-O2 chimeric constructs, three-step overlap extension PCR was performed using (i) OCIAD1-BspEI-fd and TM_1-OCIAD2-rv1 or OCIAD2-BspEI-fd and TM_2-OCIAD1-rv1 for amplification of the N-terminal sequence, (ii) TM_1-OCIAD2-fd1 and TM_1-OCIAD2-rv2 or TM_2-OCIAD1-fd1 and TM_2-OCIAD1-rv2 for the amplification of the transmembrane region, and (iii) TM_2-OCIAD1-fd2 and OCIAD1-Bam-rv or TM_1-OCIAD2-fd2 and OCIAD2-Bam-rv for the amplification of the C-terminal region. A combination of these fragments was used as template for PCR amplification and cloning into the *Bsp*EI-*Bam*HI sites of pCMV-X-FLAG to yield plasmids pCMV-TM2-OCIAD1-FLAG and pCMV-TM1-OCIAD2-FLAG.

All constructs were verified by sequencing.

### *In vitro* transcription, electroporation and infection assays

*In vitro* transcription of subgenomic replicon and full-length HCV RNA as well as electroporation were performed as described ([35] and references therein). HCVcc was produced and 50% tissue culture infective dose (TCID_50_) determined as described [37]. For the determination of intracellular infectivity, cells were harvested and subjected to three freeze and thaw cycles, followed by removal of debris by centrifugation for 2 min at 11,000 x *g*.

### Retroviral transduction

pLPCX-GFP as well as packaging constructs pMLV-NB and pMD.G, encoding Gag, Pol and VSV glycoproteins, were kindly provided by Angela Ciuffi (University of Lausanne, Switzerland) and Didier Trono (Ecole Polytechnique Fédérale de Lausanne, Switzerland), respectively. Recombinant retroviral particles were prepared in 293T cells by cotransfection of pLPCX-derived OCIAD1 constructs or pLPCX-GFP with pMLV-NB and pMD.G, as described [38].

### RNA silencing

Two siRNAs targeting human OCIAD1, designated as #1 (s29806) and #2 (s29808), as well as a non-targeted control siRNA (Silencer Select Negative Control #1) were purchased from Ambion (Life Technologies, Carlsbad, CA). Lipofectamine^®^ RNAiMAX reagent (Life Technologies) was used according to manufacturer’s recommendations, for the transfection of siRNA at a final concentration of 100 nM.

### Liver biopsies

Liver biopsy specimens used in this study have been described [21]. They were obtained from patients with chronic hepatitis C and controls in the context of routine diagnostic workup if there was sufficient material for histopathological examination and the patient’s written informed consent had been provided in accordance with the Ethics Committee of Basel.

### Confocal laser scanning microscopy

U-2 OS cells were cultured on 22-mm coverslips. The mitochondrial dye MitoTracker (Life Technologies) was added to the culture medium for 30 min, followed by fixation with 2% paraformaldehyde, permeabilization using 0.3% Triton X-100 and blocking with 3% bovine serum albumin. Cells were incubated with anti-OCIAD1 and anti-FLAG antibodies for 1 h at 20°C, followed by 1-h incubation with Alexa Fluor® 488- and 594-conjugated anti-mouse and anti-rabbit IgG secondary antibodies (Life Technologies). Coverslips were mounted in Antifade reagent (Life Technologies) and examined on a Leica SP5 confocal laser scanning microscope. Pearson’s coefficient was determined using ImageJ software.

### Immunoblot

Protein lysates were prepared and subjected to sodium dodecyl sulfate-polyacrylamide gel electrophoresis followed by immunoblot analysis, as described [22, 39].

### Quantitative RT-PCR

HCV and glyceraldehyde 3-phosphate dehydrogenase RNA levels were measured by SYBR green real-time PCR, as described [40].

### Statistical analyses

Significance values were calculated by using the unpaired t test with the GraphPad Prism 6 software package (GraphPad Software).

## Supporting information

S1 Raw images (PDF)

## Abbreviations

aa: amino acid
C4γ: complement component 4γ
DDB1: DNA damage-binding protein 1
GFP: green fluorescent protein
GPx8: glutathione peroxidase 8
HCV: hepatitis C virus
HCVcc: cell culture-derived HCV
mAb: monoclonal antibody
MAVS: mitochondrial antiviral signaling protein
NS3-4A: nonstructural protein 3-4A
OCIAD1: ovarian cancer immunoreactive antigen domain containing protein 1
RIG-I: retinoic acid-inducible gene I
SILAC: stable isotopic labeling using amino acids in cell culture
TC-PTP: T-cell protein tyrosine phosphatase
TCID_50_: 50% tissue culture infective dose
TLR3: Toll-like receptor 3
TRIF: TIR domain-containing adaptor inducing interferon-β.
