# Supplementary figures and images for: OCIAD1 is a host mitochondrial substrate of the hepatitis C virus NS3-4A protease
Source: PLoS One. 2020 Jul 22;15(7):e0236447. doi: 10.1371/journal.pone.0236447 (PMC7375614; doi:10.1371/journal.pone.0236447)

Figure 1A

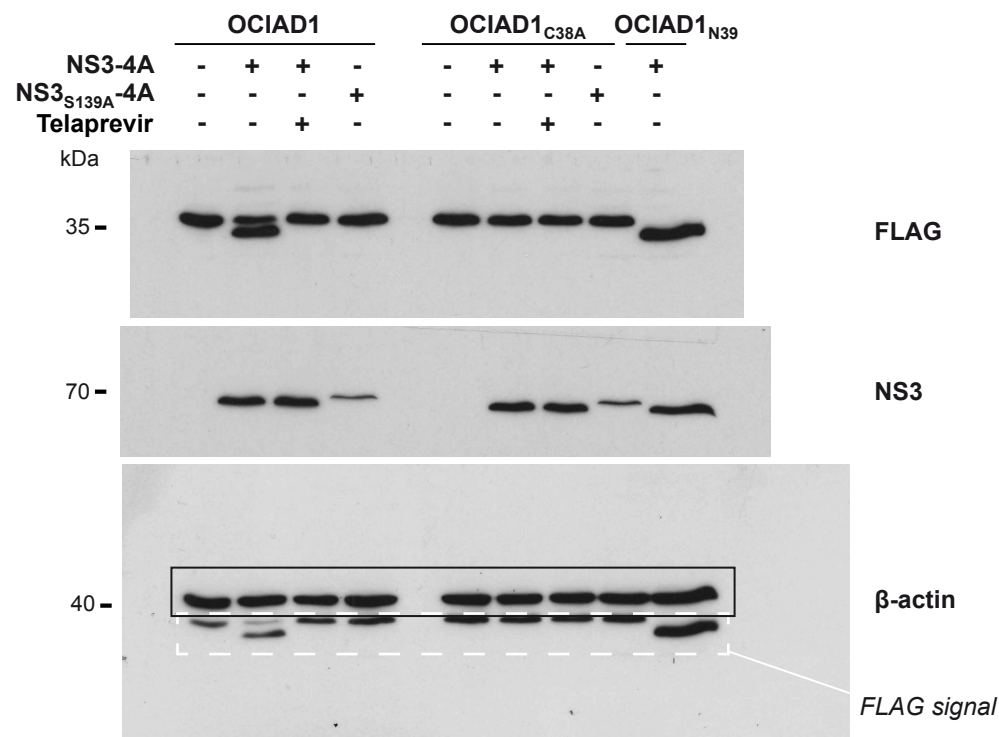

Figure 2A

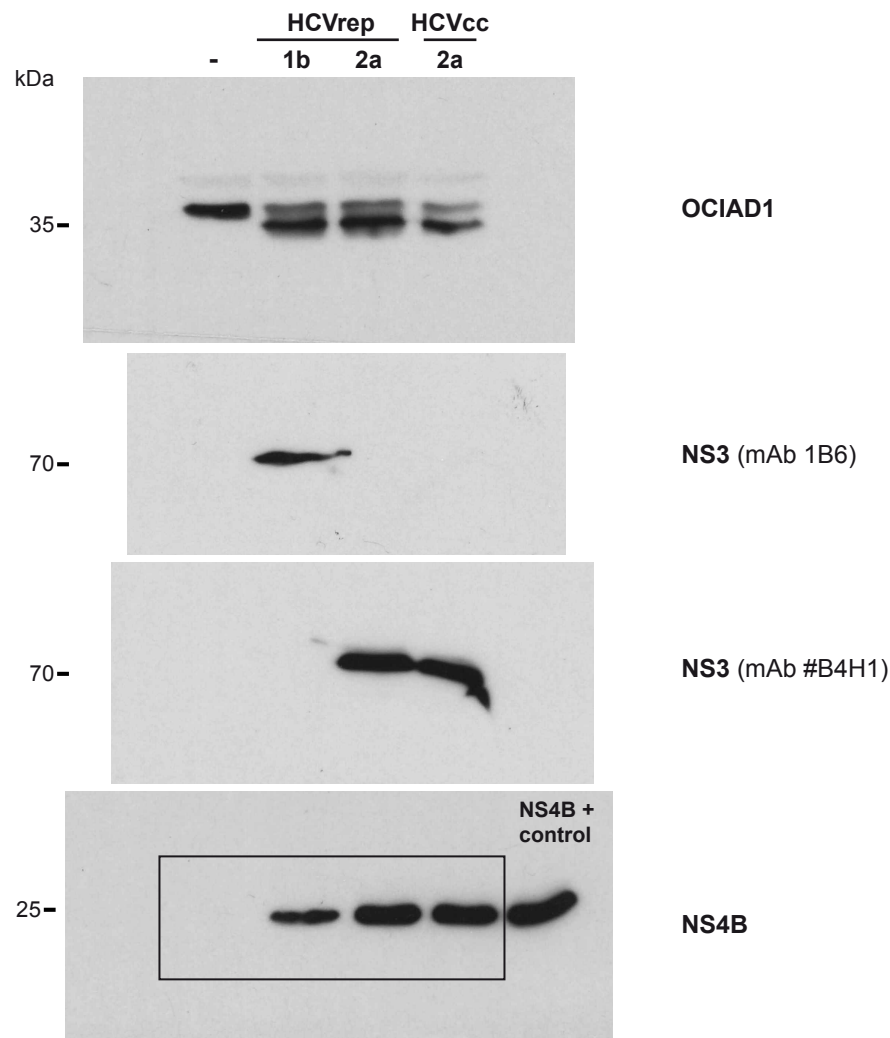

Figure 2B

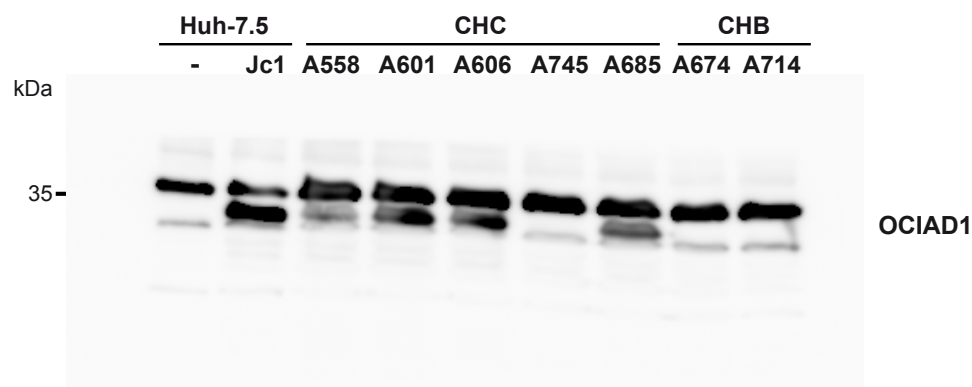

Figure 3C

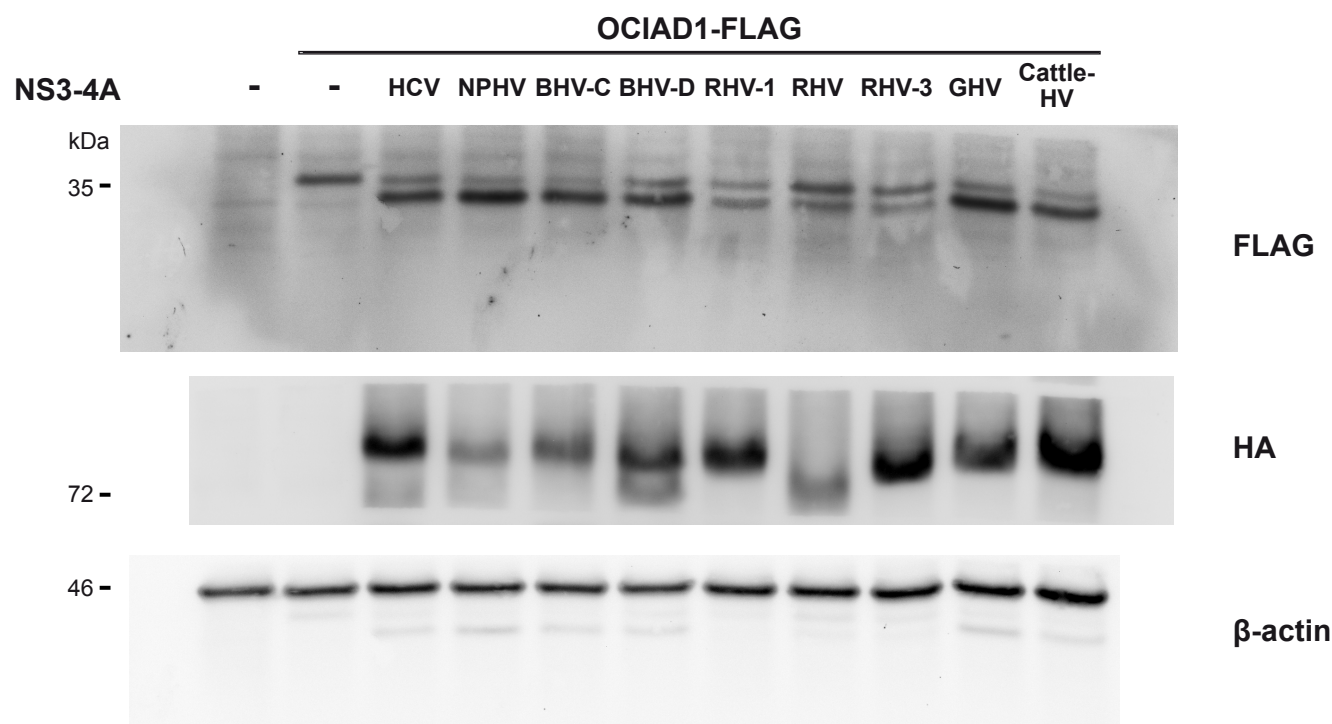

Figure 4C

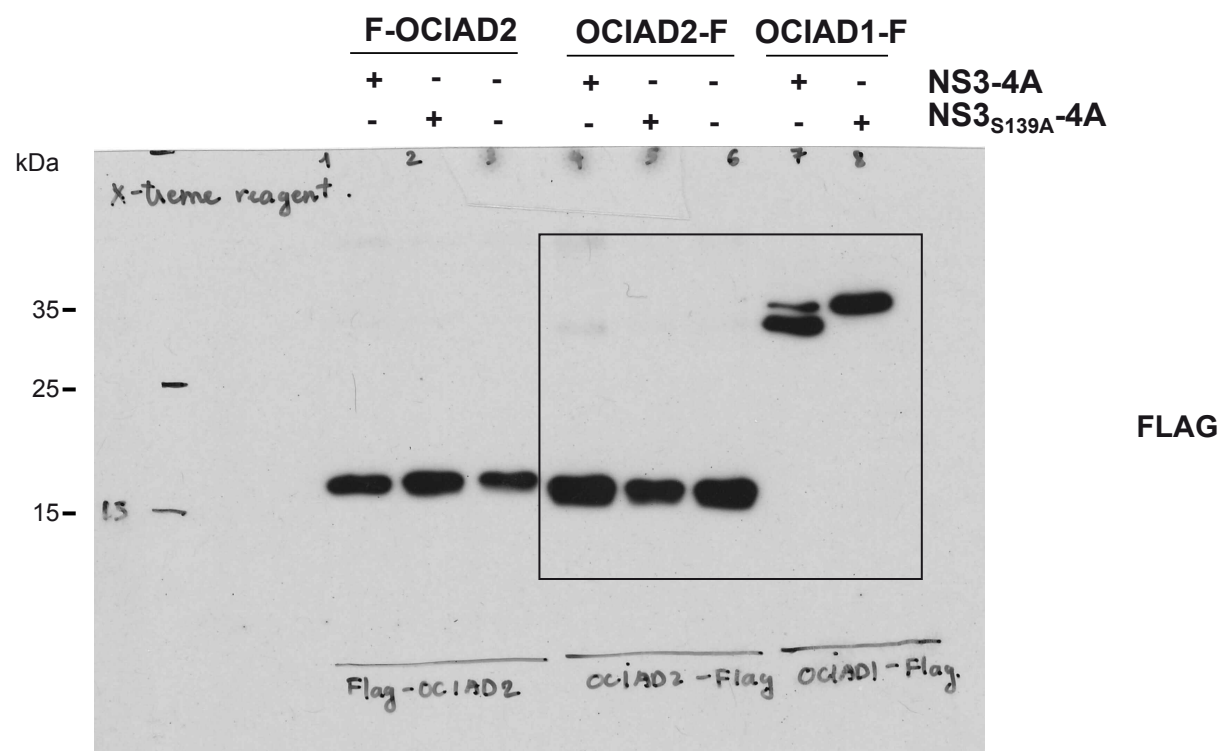

Figure 5B

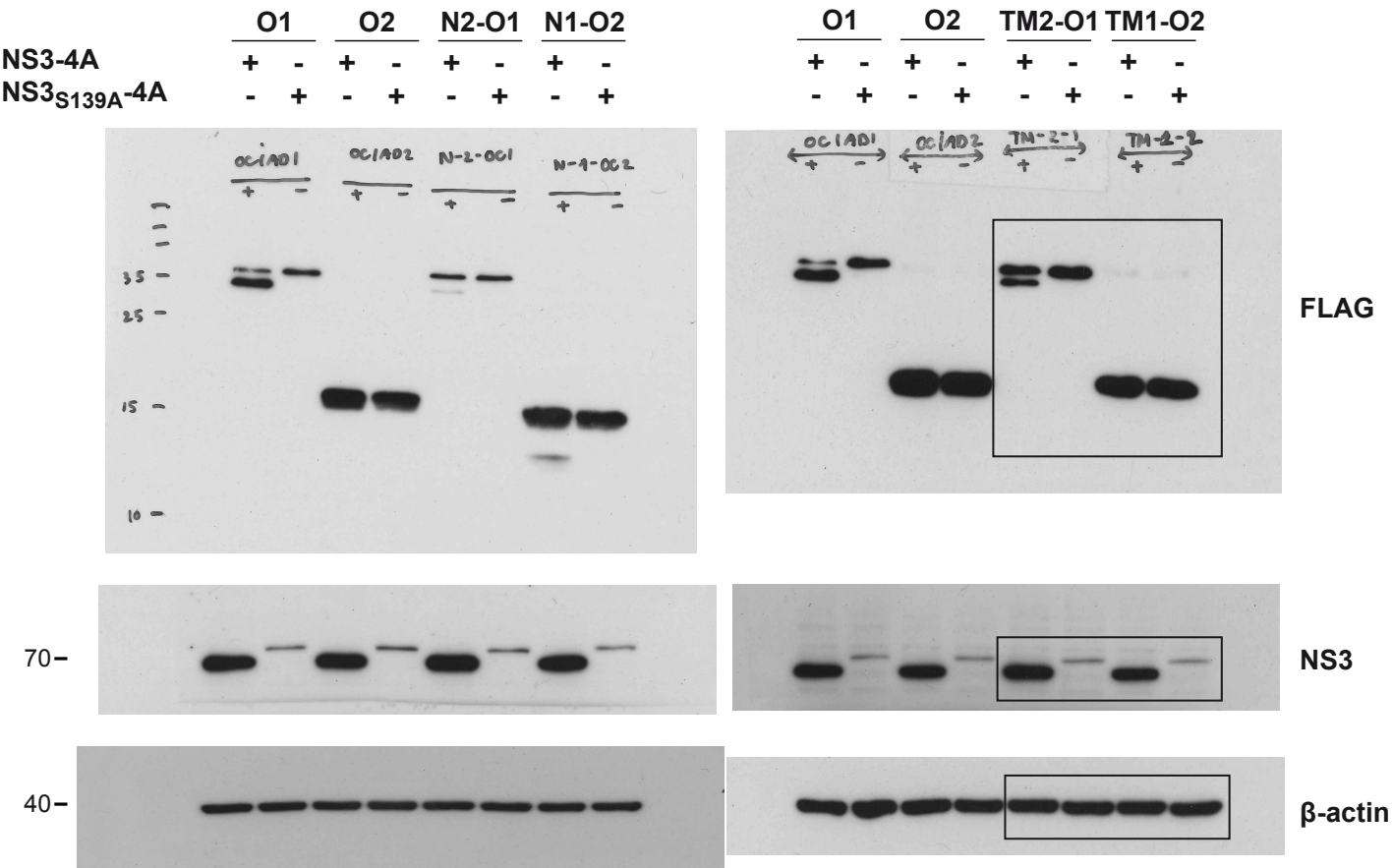

Figure 6A

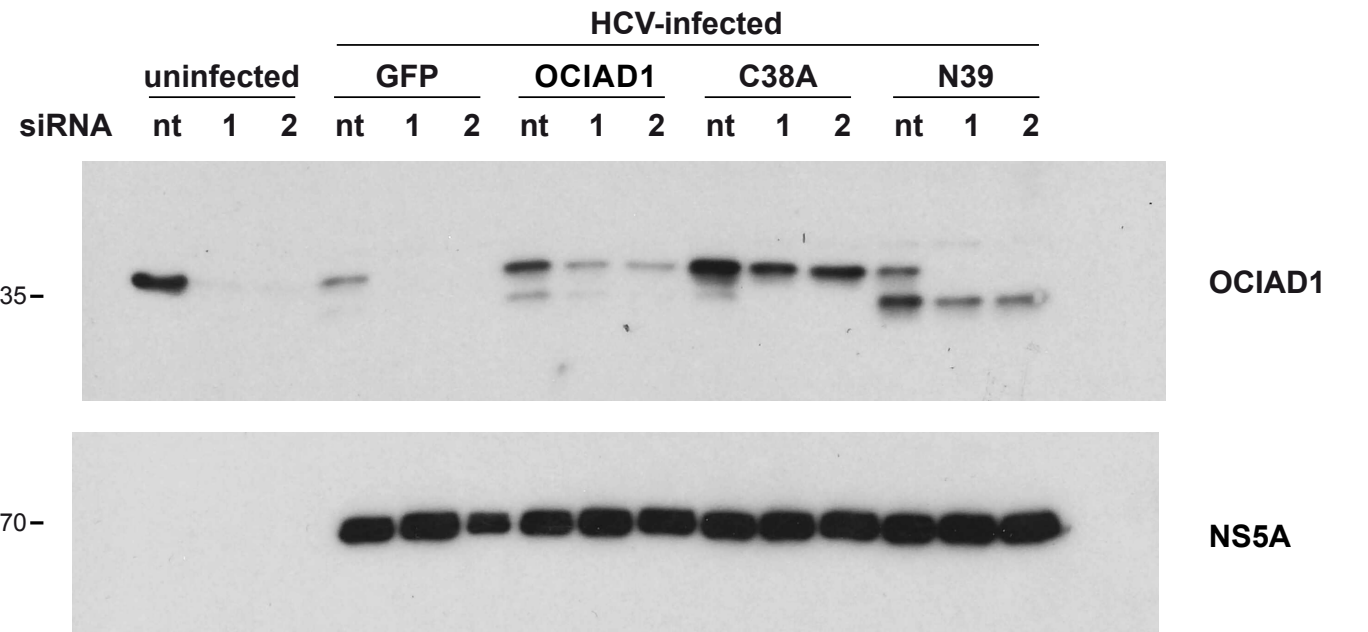

Supplement: S1 Raw images — (PDF) [file pone.0236447.s001.pdf]
